# The nasal oxygen practice in intensive care units in China: A multi-centered survey

**DOI:** 10.1371/journal.pone.0203332

**Published:** 2018-08-30

**Authors:** Zunjia Wen, Junyu Chen, Lanzheng Bian, Ailing Xie, Mingqi Peng, Mei Li, Li Wei

**Affiliations:** 1 SICU, Children’s Hospital of Nanjing Medical University, Nanjing, China; 2 Nursing Department, Children’s Hospital of Nanjing Medical University, Nanjing, China; University of Notre Dame Australia, AUSTRALIA

## Abstract

**Background:**

Nurses frequently administer nasal oxygen therapy for patients in intensive care units (ICUs). However, little is known about the current status, nurses’ management and perception on the nasal oxygen therapy in China. Therefore, we aimed to investigate the nasal oxygen practice of ICUs in China to provide insights into future direction.

**Methods:**

A cross-sectional survey on 10 hospitals was conducted. A self-designed questionnaire was administered to ICU nurses. Descriptive statistics, univariate, and multiple stepwise regression analyses were performed to analyze the respondents’ questionnaires.

**Results:**

A total of 580 respondents with a response rate of 96.67% were included in this study. The average correct answer rate was 58.28%. The current status of nasal oxygen administration in ICUs in Chinese hospitals lagged behind the recommendations of related guidelines. Nurses in China were eager to learn about the updated knowledge on oxygen therapy. The gender, age, clinical experience, degree, job title, and classification of working hospitals were not related to the oxygen therapy-related knowledge scores (all P>0.05).

**Conclusion:**

Many deficiencies are observed regarding the nasal oxygen practice in ICUs of Chinese hospitals. Increased efforts by authorities and medical staff are required to narrow the gap between the current status of oxygen practice and the recommendations from related guidelines.

## Introduction

Oxygen therapy is the most commonly used treatment in hospitals to increase the hemodynamic efficiency in patients. However, incorrect oxygen administration may lead to high risk of hypoxemia, hyperoxemia, respiratory problem, and even death [1, 2]. Therefore, the timely and appropriate oxygen administration is extremely important to patient care, particularly in intensive care units (ICUs). In ICUs, nurses can independently manage nasal oxygen therapy to prevent the detrimental effects of hypoxemia[3]. Therefore, their knowledge and strategies on nasal oxygen therapy can largely influence the management of nasal oxygen delivery for ICU patients[4].

Generally, nasal oxygen delivery is administered to nearly every ICU patient, except for mechanically ventilated (MV) patients. Several previous studies [5–7] identified many differences in oxygen management of MV patients. Nevertheless, to date, very limited studies have focused on the current status of knowledge and practice of ICU nurses on nasal oxygen practice. Nasal oxygen delivery is very common in ICU patients, and nurses’ knowledge, attitude, and oxygen therapy management may largely affect the outcomes of ICU patients[8]. Therefore, the current status of nurses’ perception and knowledge about nasal oxygen therapy warrants to be understood, and the gap between related oxygen guideline and current clinical practice needs to be clarified.

Based on literature review, we found that studies on the clinical use of oxygen therapy were very few, besides, most studies [9, 10] aimed to compare the effects and safety of different oxygen therapies. Limited studies [11, 12] analyzed the knowledge and opinion of medical staff regarding oxygen therapy. To the best of our knowledge, no study has focused on the current status of nasal oxygen therapy in ICUs in China, and the knowledge, perception, and attitude of nurses on oxygen therapy in China also remain unclear. Therefore, we conducted this multi-centered survey to analyze the current status of oxygen practice in ICUs in China, to identify the nurses’ perception and attitude toward nasal oxygen therapy, and consequently ascertain the inadequacies in nursing management and explore the underlying reasons.

## Methods

### Ethical consideration

Ethical consideration was not applicable in this study. We did not collect any individual-level identifying information in this study.

### Questionnaire

Previous study[13] reported a questionnaire on the attitudes, beliefs, and stated practices relating to the management of oxygen therapy in the ICU, but considering that the differences of China and Canada were large, and the questionnaire was designed in 1999, it was not appropriate for use in our survey, therefore, we attempted to design and validate the questionnaire by ourselves. We searched the Pubmed, EMBASE, Science Direct, China National Knowledge Infrastructure and Wanfang Database to identify related studies with keywords of “oxygen therapy” and “ICU” and (“survey” or “current status” or “opinions” or “understanding”), and reviewed a large number of related literatures on oxygen therapy to construct the structure and contents of questionnaire, and after several rounds of discussions on the survey content, we formed the first draft of questionnaire. The questionnaire included four parts: demographic characteristics of respondents, knowledge on nasal oxygen administration, current status of ICU nasal oxygen administration, and the subjective feelings of nurses on oxygen therapy. The questionnaire was reviewed and modified by 10 nursing experts with two rounds of queries, and its content validity index was 0.88. Furthermore, we randomly selected 40 ICU nurses from our hospital to fill out the questionnaire and re-fill it out after 2 weeks. The κ coefficient was in the range of 0.85–0.91, thereby indicating that the questionnaire had good retest reliability [14]. Finally, we constructed an online questionnaire at https://www.wjx.cn/, which is the largest Chinese website platform for online questionnaire survey.

### Population and sampling

The target populations for this survey were ICU nurses. We approached 10 tertiary academic hospitals with a total of 1218 ICU nurses in three provinces which may roughly represent the developed, developing and less developing areas in China, the distributed number of ICU nurses in each hospital was based on the corresponding proportion of total ICU nurses, then the nurses in each hospital were randomly selected based on the distributed number of each individual identified hospital. Finally, 600 ICU nurses were identified in this survey. Each ICU nurse was invited by WeChat to respond to the questions on the online survey site, and a single reminder letter was sent two weeks later if it’s still not answered. A response rate higher than 95% is more ideal for further data analysis[15].The responses were recorded during 1 to 5, March, 2018.

### Data analysis

All responses were shown as a percentage of the total number of responses for each particular question. We conducted descriptive analysis on the characteristics and scores of respondents. No imputation was used for data analysis because the proportion of missing values was considerably small. We conducted *t*-test, ANOVA, chi-squared test, or nonparametric Wilcoxon (Mann–Whitney) rank-sum test to detect differences between groups. We also conducted univariate and multiple stepwise regression analyses to identify the factors affecting the scores of respondents. We used SPSS 21.0 (SPSS Inc., USA) to analyze the data, and a P value <0.05 was considered statistically significant in this study, all of the tests were two-sided.

## Results

### Characteristics of respondents

We initially identified 600 potential ICU nurses for this survey and received 580 qualified answers, with an overall response rate of 96.67%. Table 1 shows the demographic characteristics of respondents. Female nurses took a large proportion of 88.79% among respondents. Generally, the nursing staff in ICU was relatively young with an age range of 20–35 years old. Moreover, most respondents had clinical experience of less than 10 years. A total of 75.86% nurses obtained bachelor’ degree, and 58.97% nurses were nursing practitioners.

**Table 1:** Basic characteristics of respondents.

Items	Nurses (n, %)	Items	Nurses (N, %)
Age, year		Gender	
<20	2 (0.34)	Female	515 (88.79)
~25	132 (22.76)	Male	65 (11.21)
~30	242 (41.72)	Clinical experience, year	
~35	141 (24.31)	<2	87 (15)
~40	44 (7.59)	~2	151 (26.03)
>40	19 (3.28)	~5	227 (39.14)
Job title		~10	70 (12.07)
Junior nurse	151 (26.03)	~15	16 (2.76)
Nurse practitioner	342 (58.97)	~20	29 (5)
Nurse-in-charge	75 (12.93)	Degree	
Vice professor of nursing	10 (1.72)	Associate	131 (22.59)
Professor of nursing	2 (0.34)	Bachelor	440 (75.86)
		Master	9 (1.55)

### Knowledge on oxygen therapy

Table 2 presents the answers of ICU nurses regarding their knowledge about oxygen therapy. The correct answer rate differed hugely among respondents with an average correct answer rate of 58.28%. However, the correct answer rate on the question that ethanol should be added into the humidified bottle to reduce alveolar surface tension and improve oxygenation for patients with acute pulmonary edema was only 0.86%, which was significantly lower than those of other questions.

**Table 2:** Answer distribution of questions regarding the knowledge on oxygen therapy.

Questions	Correct answers (n, %)	Incorrect answers (n, %)
Is oxygen soluble in water at room temperature (18°C–30°C)?	237 (40.86)	343 (59.14)
What is the general oxygen concentration in air?	517 (89.14)	63 (10.86)
Generally, to achieve the effect of oxygen inhalation, the inhaled oxygen concentration should not be less than what concentration?	117 (20.17)	463 (79.83)
When the inhaled oxygen flow is 3 L/min, what is the inhaled oxygen concentration?	520 (89.66)	60 (10.34)
High oxygen flow is defined as the oxygen flow higher than?	486 (83.79)	94 (16.21)
For newborns and premature infants, the oxygen concentration should be less than?	231 (39.83)	349 (60.17)
For patients with acute pulmonary edema, what kind of ethanol should be added to the humidified bottle to reduce alveolar surface tension and improve oxygenation?	5 (0.86)	575 (99.14)
Which of the following items is not a complication of oxygen practice?	222 (38.28)	358 (61.72)
To ensure the safety of oxygen therapy, four prophylaxes are promoted, excluding?	555 (95.69)	25 (4.31)
If the patient shows insignificant improvement in hypoxia after nasal oxygen administration, what should you do first?	490 (84.48)	90 (15.52)

### Current status of nasal oxygen administration in ICU

**Fig 1:**
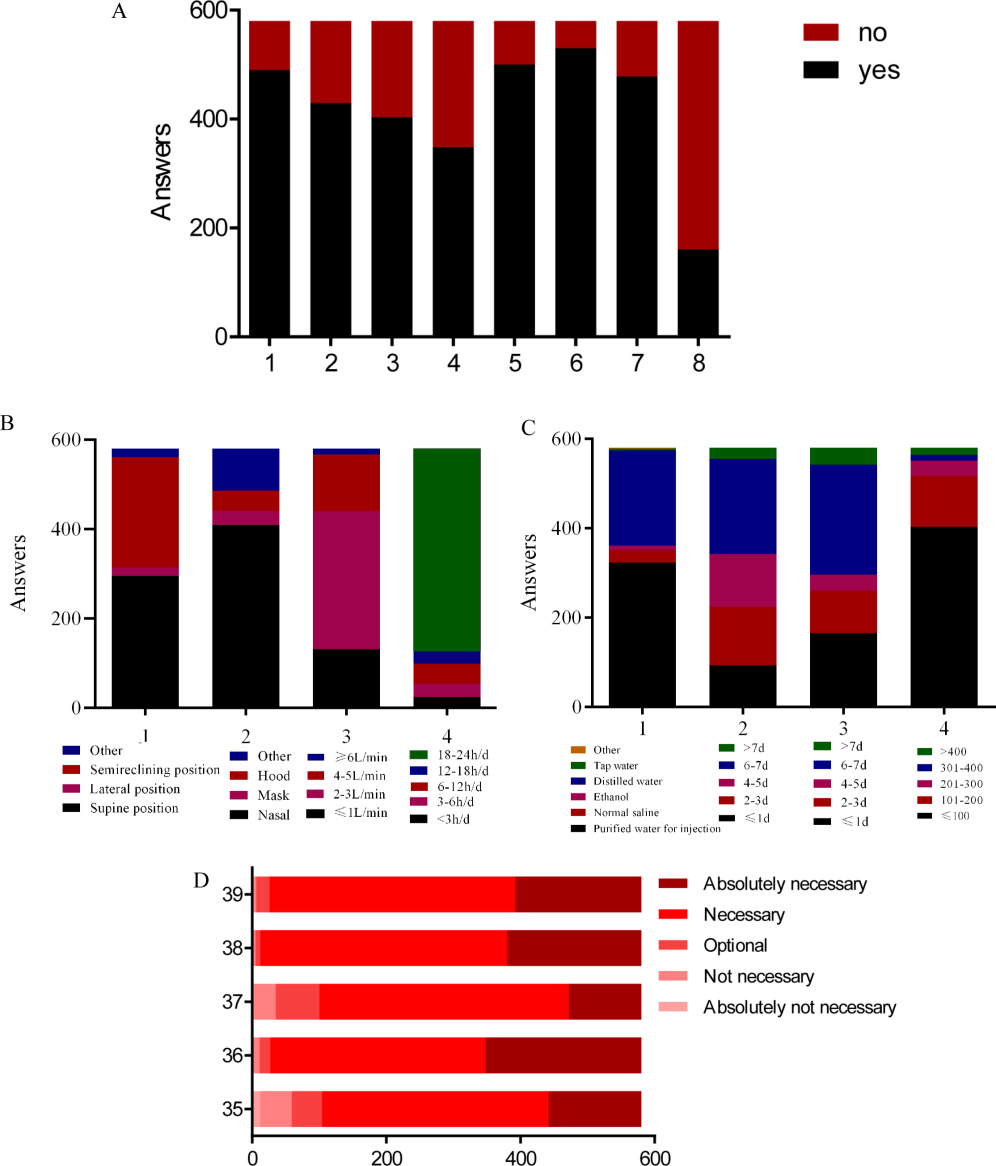
Answer distribution on the current status and nurses’ perception regarding oxygen administration in ICU. Notes: A: 1, Is it common to use a humidified bottle in humidifying the inhaled nasal oxygen regardless of oxygen flow?; 2, Is the humidifying bottle routinely used in ICU disposable?; 3, Will your ICU perform bacteriological tests on nasal oxygen delivery devices?; 4, Will you disinfect the central oxygen terminal oxygen hub regularly?; 5, Should the patient’s comfort during oxygen administration be assessed?; 6, Will you evaluate the patient’s nasal mucosa after oxygen inhalation?; 7, Will your nursing staff be trained specifically for nasal oxygen therapy?; 8, Have you met patient who refused oxygen therapy? B: 1, What is the most common oxygen intake position in your ICU?; 2, What is the most common method of administering oxygen in your ICU?; 3, What is the most common nasal oxygen flow in your ICU?; 4, What is the approximate oxygen intake time per patient?; C: 1, What is the fluid commonly used in your ICU humidifier bottle?; 2, How often do you change the humidified bottle?; 3, How often do you change the nasal oxygen catheter?; 4, What is the average cost of nasal oxygen device (humidified bottle, oxygen catheter) for each patient in your ICU?; D: 35, Do you think it is necessary to humidify the oxygen in medium or low flow?; 36, Do you think it is necessary to precisely regulate the flow and concentration of inspired oxygen?; 37, Do you think it is necessary to regulate the temperature of inhaled oxygen?; 38, To ensure the effect of oxygen administration, should you care about the patient’s perception about the practice?; 39. Do you think it is necessary to establish oxygen-related nursing guidelines and update the related evidences?.

As shown in Fig 1A, 84.48% of the respondents reported that they routinely used humidified bottle to humidify the inhaled nasal oxygen regardless of the oxygen flow, and the humidified bottle routinely used in ICU was disposable. Furthermore, 69.48% of the respondents indicated that they conducted bacteriological tests on nasal oxygen delivery devices, and only 60% would disinfect the central oxygen terminal regularly. Most respondents evaluated patient’s comfort and nasal mucosa during oxygen administration. Nonetheless, only 27.59% of them had been specifically trained for nasal oxygen therapy.

Fig 1B and 1C illustrate the relative proportion of the details on nasal oxygen administration of respondents in ICUs. Briefly, supine position was the most commonly used nasal oxygen intake position (50.86%), and the most common method to delivery oxygen was the nasal route (70.52%). In addition, 2–3 L/min was the generally used oxygen flow for nasal oxygen administration (53.28%), and most patients received long oxygen administration for 18–24 hours out of total ICU stay length (78.28%). Purified water for injection was the most commonly used in humidified bottle (56.73%), and the humidified bottle (36.69%) and nasal oxygen catheter (40.24%) were changed every 6–7 days. Furthermore, the average cost of nasal oxygen device for each patient was less than 100 RMB (approximately 11.4 dollars).

### Nurses’ perception about oxygen practice

Fig 1D reveals the nurses’ perception about oxygen practice in ICU. Most nurses (82.07%) reported that it’s necessary to humidifying oxygen routinely even in medium or low flow to regulate the flow, concentration (95.34%), and temperature (82.76%) of inhaled oxygen precisely. In addition. 97.93% respondents reported that the patients’ feeling during oxygen administration should be considered. Nearly all respondents (98.97%) also believed that establishing oxygen-related nursing guidelines and updating related evidence were essential.

### Factors affecting the oxygen therapy-related knowledge score

As shown in Table 3, the gender, age, clinical experience, degree, job title, and classification of working hospitals were not related to the oxygen therapy-related knowledge scores (all P>0.05).

**Table 3:** Multiple regression analysis on the factors affecting the oxygen therapy-related knowledge scores.

Independent variables	Dependent variable	Unstandardized regression coefficient (standard error)	t	P	F	Adjusted R square
	Correct answer score	1.903 (0.326)	5.839	0.000	0.920	−0.001
Gender		0.122 (0.102)	1.202	0.230		
Age		0.074 (0.066)	1.120	0.263		
Clinical experience		0.010 (0.053)	0.195	0.845		
Degree		−0.067 (0.083)	-0.802	0.423		
Job title		−0.007 (0.053)	-0.138	0.890		
Classification of hospitals the respondents work for		0.063 (0.100)	0.634	0.526		

## Discussion

Oxygen administration increase the amount of oxygen in plasma to improve the oxygen supply in tissues, thereby promoting the metabolism of body’s internal environment [16]. A number of clinical studies [17–19] have shown that oxygen inhalations play an important role in disease rehabilitation. Particularly, continuous low-flow oxygen therapy produces a good therapeutic effect on the symptoms of chronic respiratory failure caused by various diseases; it also has been taken as the primary treatment option in improving chronic hypoxia[20]. To the best of our knowledge, only very few related studies have provided insights into this issue, and to date no Chinese study has been conducted to address it. Previous study[21] investigated the intensivists’ opinion and self-reported practice of oxygen therapy, and concluded that recognizing the factors influencing oxygen administration decisions is necessary to the potential conduct of interventional studies, as well as for the development of improved guidance for oxygen therapy in critical care. Another survey [22] on oxygen delivery practices in UK pediatric ICUs outlined a clinical scenario with subsequent questions on oxygenation targets for five common diagnoses observed in critically ill children, found that PaO_2_ targets were not commonly used in clinical settings. This study focused on the basal, physiological, chemical properties and knowledge about oxygen, oxygen therapy and nasal oxygen administration, found that nurses’ knowledge on oxygen administration in China was generally not accepted, and the current status of oxygen administration in China largely differed from the practice recommended by related guidelines. Although nurses were eager to learn about the updated knowledge on oxygen therapy, the resources in China were largely limited. Therefore, much efforts are needed to improve the nursing quality of administering oxygen therapy.

To determine the nurses’ understanding on the knowledge of oxygen therapy, we searched several Chinese-related guidelines[23, 24] and the latest nursing course book in China to identify the items of questionnaire. We also consulted many clinical nursing experts to ensure that the contents conferred to the clinical practice. Specifically, according to a course book[25] for nursing student in China, 20%–30% ethanol should be added to the humidified bottle of patients with acute pulmonary edema to reduce alveolar surface tension and improve oxygenation. Nevertheless, we found no related recommendation on this issue in any foreign guidelines. The updated book knowledge lags behind the guideline recommendations in China, resulting in that nursing students are learning outdated contents, which should be taken into discreet consideration by nursing authority in China.

Any innovation should be based on the understanding of current status. Our survey indicated that supine position was the most common oxygen intake position, and nasal route was the most common method of supplying oxygen. Furthermore, nasal oxygen was given nearly all day long (18–24 h) with low flow (2–3 mL/min). Current guidelines[8, 26, 27] and evidence[28, 29] have supported that humidifying the inhaled nasal oxygen in the administration of low flow oxygen is unnecessary, given its ineffectiveness on humidification and potential for bacterial contamination[30]. However, inhaled nasal oxygen is routinely humidified regardless of the oxygen flow used in China. The use of disposable humidified bottle can increase the financial burden of patients. To the best of our knowledge, although several related Chinese studies [31–33] have reported that humidifying oxygen routinely is unnecessary in the low-flow delivery of oxygen, updated recommendations in related guidelines and course books are lacking in China, the knowledge should be updated in the future as soon as possible.

Nurses’ perception about oxygen practice in ICU is notable. A large proportion of respondents believed that we should put more attentions on the flow, concentration, and temperature of inhaled oxygen. The patients’ feelings during oxygen administration should also be considered, thereby indicating that the population of nurses is fraternal, enterprising, and assiduous. Nonetheless, very few nurses had been specifically trained for nasal oxygen therapy. Thus, policy-oriented, high-level design should be developed in China, The nasal oxygen administration training should also be given considerable attention, meanwhile, oxygen-related nursing guidelines should also be established and updated in China.

Several limitations in this study should be considered. Firstly, the questionnaire in this study is based on our understanding about the condition of ICUs in Chinese hospitals and related literature review, and this study is limited to Chinese health setting. Therefore, the content bias may exist. Nevertheless, we revised and verified the contents with nursing expert consultation and pre-survey. Secondly, we can’t exclude the possibility of response bias because we conducted this online survey with assistance from the nursing heads from different hospitals. Thirdly, we didn’t find any relationship between gender, age, clinical experience, degree, job title, and classification of working hospitals and the oxygen therapy-related knowledge scores, which may be explained by the limited number of respondents, our respondents only came from 10 hospitals in three provinces, population larger than that of present study with extensive regions should be included in future studies. This study also shows several strengths. The response rate in our online survey was 96.67%, which limits the effect of no-response bias. To our knowledge, this work is the first study on oxygen therapy practices in China to date, our results provide further insights into the reform of nursing field in China. On the basis of this survey results, we are conducting and highlighting several follow-up studies regarding guideline establishment and update practice on nasal oxygen therapy.

In conclusion, the current status of nasal oxygen administration in the ICUs of Chinese hospitals is unsatisfactory, the ICU nurses in China are eager to embrace new and substantial evidences in this area. Considering the generality and importance of oxygen administration, more efforts should be put into the reform of nursing management and update of related knowledge and guidelines on oxygen therapy in the future.

## Supporting information

S1 File The questionnaire in Chinese. (DOCX)

S2 File The questionnaire in English. (DOCX)

S3 File PLOSOne_clinical_studies_checklist. (DOCX)

S4 File STROBE_checklist_v4_combined_PlosMedicine. (DOCX)

S5 File Language editing certificate. (PDF)
