# Supplementary material for: The nasal oxygen practice in intensive care units in China: A multi-centered survey
Source: PLoS One. 2018 Aug 30;13(8):e0203332. doi: 10.1371/journal.pone.0203332 (PMC6117075; doi:10.1371/journal.pone.0203332)
Supplement: S1 File — (DOCX) [file pone.0203332.s001.docx]

**儿科重症护理单元(ICU)给氧现状调查**

**第一部分 一般情况调查**

本部分旨在了解您的一般个人情况，所有信息将严格保密，请如实填写。

1、您所在的医院：

2.您的性别：

- 男
- 女

3、您的年龄（周岁）：

4、您从事护理工作的年限（年）：

5、您的学历为：

- 大专及以下
- 本科
- 研究生及以上

6、您目前的职称：

- 护士
- 护师
- 主管护师
- 副主任护师
- 主任护师

**第二部分 给氧相关知识调查**

本部分旨在调查您对给氧相关知识的了解情况，请根据您的理解作出选择。

9、氧气在室温（18-30℃）下是否易溶于水？

- 是
- 否
- 不清楚

10、正常空气中的氧浓度大约为

- 12%
- 21%
- 30%
- 45%

11、为达到氧气吸入的效果，吸入氧气的浓度不应低于

- 20%
- 25%
- 29%
- 35%
- 42%

12、当患儿的吸入氧流量为3L/min时，其吸入氧浓度为：

- 21%
- 25%
- 29%
- 33%
- 37%

13、当吸入氧流量大于多少L/min时即为高流量吸氧？

- 1L/min
- 2L/min
- 3L/min
- 4L/min
- 5L/min
- 6L/min

14、对于新生儿、早产儿不应长时间高浓度给氧，其氧浓度应低于多少？

- 30%
- 40%
- 50%
- 60%

15、对于急性肺水肿的患者，应在湿化瓶内加入何种浓度的乙醇以降低肺泡表面张力，改善患者氧合功能？

- 10-20%乙醇
- 20-30%乙醇
- 30-40%乙醇
- 40-50%乙醇
- 50-60%乙醇

16、以下哪项不是临床上给氧的不良并发症？

- 氧中毒
- 鼻粘膜干燥
- 肺不张
- 视网膜病变
- 呼吸兴奋

17、为保证用氧安全，应当做好“四防”，但不包括以下哪项？

- 防火
- 防热
- 防震
- 防水
- 防油

18、如果患者用氧后，其缺氧状况无明显改善，您首先应当如何处理？

- 上调氧流量
- 使用呼吸兴奋剂
- 检查吸氧装置是否连接完好
- 通知医生
- 行机械通气治疗

**第三部分 ICU给氧情况调查**

本部分针对您所在ICU的给氧现状进行调查，选项并无对错之分，请根据您的实际情况选择，请勿漏选。

19.您所在ICU最为常见的吸氧体位为：

- 仰卧位
- 侧卧位
- 半坐位
- 其他：

20、您所在ICU最为常见的吸氧方式为：

- 鼻导管
- 面罩
- 头罩
- 其他

21、您所在ICU平均每位患儿的吸氧时间约为：

- ≤3h/d
- ≤6h/d
- ≤12h/d
- ≤18h/d
- ≤24h/d

22、您所在ICU最为常见的鼻导管给氧流量为：

- ≤1 L/min
- ≤2 L/min
- ≤3 L/min
- ≤4 L/min
- ≤5 L/min
- ≤6 L/min
- ＞6 L/min

23、您所在ICU进行鼻导管给氧时，无论吸氧流量的大小，是否常规使用湿化瓶对吸入氧气进行气泡湿化？

- 是
- 否

24、您所在ICU常规使用的湿化瓶是否为一次性包装使用的湿化瓶？

- 是
- 否

25、您所在ICU湿化瓶内常用的液体为

- 注射用水
- 生理盐水
- 乙醇
- 蒸馏水
- 自来水

26、您所在ICU是否会对吸氧装置（湿化瓶、氧导管）的进行细菌学检测？

- 是
- 否

27、您所在ICU常规多久更换一次吸氧用的湿化瓶？

- ≤1天
- ≤2天
- ≤3天
- ≤4天
- ≤5天
- ≤6天
- ≤7天
- ＞7天

28、您所在ICU常规多久更换一次吸氧导管？

- ≤1天
- ≤2天
- ≤3天
- ≤4天
- ≤5天
- ≤6天
- ≤7天
- ＞7天

29、您所在ICU是否会对中心供氧终端氧气插孔进行消毒？

- 是
- 否

30、您所在ICU平均每位患者的吸氧装置（湿化瓶、氧导管）的花费大约为

- ≤100元
- 101-200元
- 201-300元
- 301-400元
- >400元

31、您所在ICU给氧时是否会评估患者的吸氧舒适度？

- 是
- 否

32、您所在ICU给氧后是否会评估患者的鼻粘膜情况？

- 是
- 否

33、您所在ICU是否针对给氧疗法进行过专门的培训?

- 是
- 否

34、您所在ICU是否遇到孩子拒绝氧疗的情况，如果是，请注明原因。

- 是
- 否

**第四部分 护理人员对氧疗相关主观感受调查**

本部分选用Likert 5级分级法，分值1到5代表从“完全没必要”到“完全有必要”，旨在了解您对氧疗的一些相关体验，请根据您的实际情况，选择最符合您主观感受的选项，请勿漏选。

35、 您认为有无必要进行中低流量给氧的湿化？

- 完全没必要
- 没必要
- 可有可无
- 有必要
- 完全有必要

36、您认为有无必要精确调控吸入氧气的流量和浓度？

- 完全没必要
- 没必要
- 可有可无
- 有必要
- 完全有必要

37、您认为有无必要对吸入氧气的温度进行调控？

- 完全没必要
- 没必要
- 可有可无
- 有必要
- 完全有必要

38、为保证给氧的效果，您认为有无必要在意患者的给氧舒适度？

- 完全没必要
- 没必要
- 可有可无
- 有必要
- 完全有必要

39、您认为有无必要建立氧疗相关的护理指南并进行证据更新？

- 完全没必要
- 没必要
- 可有可无
- 有必要
- 完全有必要
