# Supplementary material for: The nasal oxygen practice in intensive care units in China: A multi-centered survey
Source: PLoS One. 2018 Aug 30;13(8):e0203332. doi: 10.1371/journal.pone.0203332 (PMC6117075; doi:10.1371/journal.pone.0203332)
Supplement: S2 File — (DOCX) [file pone.0203332.s002.docx]

**Investigation on the oxygen practice in intensive care unit (ICU)**

**Part I The general information of respodents**

This section is intended to understand your general personal situation, all information will be strictly confidential, please fill out the truth.

1. Your hospital:

2. Gender：

- Male
- Female

3、Age：

4、Working experience：

5、Academic degree：

- Associate
- Bachelor
- Master

6、Job title:

- Junior nurse
- Nurse practitioner
- Nurse-in-charge
- Vice professor of nursing
- Professor of nursing

**Part II Investigation on the oxygen related knowledge**

This section is designed to investigate your oxygen-related knowledge. Please choose according to your understanding.

9、Is oxygen soluble in water at room temperature (18 °C–30 °C)?

- yes
- no
- unclear

10、What is the general oxygen concentration in air?

- 12%
- 21%
- 30%
- 45%

11、Generally, to achieve the effect of oxygen inhalation, the inhaled oxygen concentration should not be less than what concentration?

- 20%
- 25%
- 29%
- 35%
- 42%

12、When the inhaled oxygen flow is 3 L/min, what is the inhaled oxygen concentration?

- 21%
- 25%
- 29%
- 33%
- 37%

13、High oxygen flow is defined as the oxygen flow higher than ?

- 1L/min
- 2L/min
- 3L/min
- 4L/min
- 5L/min
- 6L/min

14、For newborns and premature infants, the oxygen concentration should be less than?

- 30%
- 40%
- 50%
- 60%

15、For patients with acute pulmonary edema, what kind of ethanol should be added to the humidified bottle to reduce alveolar surface tension and improve oxygenation?

- 10-20% ethanol
- 20-30% ethanol
- 30-40% ethanol
- 40-50% ethanol
- 50-60% ethanol

16、Which of the following items is not a complication of oxygen practice?

- Oxygen poisoning
- Dry nasal mucosa
- Lung infidelity
- Retinopathy
- Breathing excitement

17、To ensure the safety of oxygen therapy, four prophylaxes are promoted, excluding what?

- Fire prevention
- Heat prevention
- Shock prevention
- Water prevention
- Oil prevention

18、If the patient shows insignificant improvement in hypoxia after nasal oxygen administration, what should you do first?

- Up-regulate oxygen flow
- Use respiratory stimulants
- Check if the oxygen absorber is connected properly
- Notify the doctor
- Mechanical ventilation treatment

**Part III** The ICU oxygen practice

This section investigates the current status of oxygen supply in your ICU. There is no right or wrong option. Please choose according to your actual situation.

19. The most common oxygen inhalation position in your ICU is:

- Supine position
- Side position
- Half-sit position
- Other:

20、The most common oxygen inhalation method in your ICU is:

- Nasal catheter
- Mask
- Hood
- Other

21、The average oxygen inhalation time per patient in your ICU is approximately:

- ≤3h/d
- ≤6h/d
- ≤12h/d
- ≤18h/d
- ≤24h/d

22、What is the most common nasal oxygen flow in your ICU?

- ≤1 L/min
- ≤2 L/min
- ≤3 L/min
- ≤4 L/min
- ≤5 L/min
- ≤6 L/min
- ＞6 L/min

23、When performs nasal oxygen therapy, regardless of the size of the oxygen flow, will you use the humidification bottle to humidify the inhaled oxygen?

- yes
- no

24、Is the humidification bottle that is routinely used in your ICU is disposable packaging?

- yes
- no

25、The fluid commonly used in your ICU humidification bottle is:

- Water for injection
- Saline
- Ethanol
- Distilled water
- Tap water
- Other (please specify):

26、Does your ICU perform bacteriological tests on oxygen delivery devices( bottles, oxygen tubes)?

- yes
- no

27、How often do you change the humidification bottle for oxygen in your ICU?

- ≤1day
- ≤2day
- ≤3day
- ≤4day
- ≤5day
- ≤6day
- ≤7day
- ＞7day

28、How often do you change the oxygen inhalation catheter in your ICU?

- ≤1day
- ≤2day
- ≤3day
- ≤4day
- ≤5day
- ≤6day
- ≤7day
- ＞7day

29、Will you disinfect the central oxygen supply terminal hub?

- Yes
- No

30、The average cost for oxygen delivery device for each patient in your ICU is approximately

- ≤100RMB
- 101-200 RMB
- 201-300 RMB
- 301-400 RMB
- >400 RMB

31、Will you assess the patient's comfort when oxygen is administered?

- yes
- no

32、Will you assess the nasal mucosa when oxygen is administered?

- yes
- no

33、Has you been specially trained for oxygen therapy?

- yes
- no

34、Have you met some patient refuse to oxygen delivery?

- yes
- no

**Part IV The Subjective Feelings of Nursing Staff on Oxygen Therapy**

This section uses the Likert 5 classification method. The scores from 1 to 5 represent “completely unnecessary” to “completely necessary”. In order to understand some related experiences of oxygen therapy, please choose the most suitable one according to your actual situation.

35、Do you think it is necessary to humidify the oxygen in medium or low flow?

- Completely unnecessary
- Unnecessary
- It depends
- Necessary
- Completely necessary

36、Do you think it is necessary to precisely regulate the flow and concentration of inspired oxygen?

- Completely unnecessary
- Unnecessary
- It depends
- Necessary
- Completely necessary

37、Do you think it is necessary to regulate the temperature of inhaled oxygen?

- Completely unnecessary
- Unnecessary
- It depends
- Necessary
- Completely necessary

38、To ensure the effect of oxygen administration, should you to care about the patient’s perception about the practice?

- Completely unnecessary
- Unnecessary
- It depends
- Necessary
- Completely necessary

39、Do you think it is necessary to establish oxygen-related nursing guidelines and update the evidence?

- Completely unnecessary
- Unnecessary
- It depends
- Necessary
- Completely necessary
