# Supplementary material for: The nasal oxygen practice in intensive care units in China: A multi-centered survey
Source: PLoS One. 2018 Aug 30;13(8):e0203332. doi: 10.1371/journal.pone.0203332 (PMC6117075; doi:10.1371/journal.pone.0203332)
Supplement: S5 File — (PDF) [file pone.0203332.s005.pdf]

KG Support Limited

www.kgsupport.com
info@kgsupport.com
Phone/Fax +852 3018 1216

RECEIPT

RECEIPT #15852
DATE: July 28,2018

To:

Children's Hospital of Nanjing Medical University

Date	DESCRIPTION	Price per Page	No. of Pages	Total Amount
July 28, 2018	English Review and Editing Service			RMB 3850.00
			**Amount Received:**	**RMB3850.00**

******************************THANK YOU FOR USING OUR SERVICE!******************************
